# Transcriptional Activators of Human Genes with Programmable DNA-Specificity

**DOI:** 10.1371/journal.pone.0019509

**Published:** 2011-05-19

**Authors:** René Geiβler, Heidi Scholze, Simone Hahn, Jana Streubel, Ulla Bonas, Sven-Erik Behrens, Jens Boch

**Affiliations:** 1 Section Microbial Biotechnology, Institute of Biochemistry and Biotechnology, Martin-Luther-University Halle-Wittenberg, Halle (Saale), Germany; 2 Institute of Biology, Department of Genetics, Martin-Luther-University Halle-Wittenberg, Halle (Saale), Germany; Michigan State University, United States of America

## Abstract

TAL (transcription activator-like) effectors are translocated by *Xanthomonas* bacteria into plant cells where they activate transcription of target genes. DNA target sequence recognition occurs in a unique mode involving a central domain of tandem repeats. Each repeat recognizes a single base pair in a contiguous DNA sequence and a pair of adjacent hypervariable amino acid residues per repeat specifies which base is bound. Rearranging the repeats allows the design of novel TAL proteins with predictable DNA-recognition specificities. TAL protein-based transcriptional activation in plant cells is mediated by a C-terminal activation domain (AD). Here, we created synthetic TAL proteins with designed repeat compositions using a novel modular cloning strategy termed “Golden TAL Technology”. Newly programmed TAL proteins were not only functional in plant cells, but also in human cells and activated targeted expression of exogenous as well as endogenous genes. Transcriptional activation in different human cell lines was markedly improved by replacing the TAL-AD with the VP16-AD of herpes simplex virus. The creation of TAL proteins with potentially any desired DNA-recognition specificity allows their versatile use in biotechnology.

## Introduction

Transcription activator-like (TAL) effectors include key virulence factors of *Xanthomonas* that bind to promoter regions of plant genes and act as DNA sequence-specific transcriptional activators [1], [2], [3], [4]. As a typical feature, TAL effectors contain a central domain of tandem repeats (1 to 33.5 repeats of typically 34 amino acids) [1]. First shown for the archetype TAL effector AvrBs3, this repeat domain is essential for DNA-binding [5], [6] and represents a novel, modular type of DNA-binding domain [5]. One repeat corresponds to one DNA base pair, and the specificity of each repeat is encoded by two hypervariable amino acids (position 12 and 13) per repeat, also termed repeat-variable diresidue (RVD) [7], [8]. The last repeat contains only the first 20 conserved residues including the RVDs and is referred to as a half repeat. Each repeat functions neighbor-independently, and the linear order of repeats defines the matching DNA-sequence. In addition, the target box is extended by a 5′ T [1], [2], [7], [8]. Based on the repeat-specificity code, the target DNA specificities of several TAL effectors were correctly predicted [7], [9], [10]. While the number of repeats greatly varies in TAL effector family members, at least 10.5 repeats are required for maximal activity [7]. Accordingly, TAL effectors with different numbers of repeats (e.g. Hax2, 21.5 repeats and Hax3, 11.5 repeats) [11] show comparable transcriptional activation in reporter assays [7]. The modular architecture, a hallmark of the TAL protein repeat domain, enables simple rearrangements of desired repeat orders. Thus, TAL proteins with novel and clearly predictable DNA-recognition specificities can be constructed [7], [12], [13], [14], [15], [16], [17].

The possibility of generating proteins with programmed DNA-binding specificity is an exciting avenue to targeted genome editing and gene regulation. For these purposes, zinc finger (ZF) proteins that contain an array of ZFs targeting a given DNA sequence are already in use [18], [19]. Specifically, ZF-nucleases representing fusions between ZF proteins and the nuclease domain of the restriction enzyme FokI were applied to induce insertions and deletions at specific sites in complex genomes [19], [20]. Compared to ZF proteins, the DNA-binding specificities of TAL proteins are considerably easier to predict [1], [19]. Thus, TAL-nucleases were recently generated that cut specific DNA sites [12], [13], [14], [21], [22]. During preparation of this manuscript, initial studies showed that TAL protein derivatives can induce the expression of human genes [14], [17].

TAL effector-mediated transcriptional activation requires the C-terminal region of the protein. This region was suggested to take on the role of transcription activation as it shows similarities to acidic transcriptional activation domains (ADs) [23], [24], [25]. So far, this notion was supported by infection experiments with *Xanthomonas* strains delivering TAL effectors where the C-terminus was deleted or substituted by the AD of the herpes simplex virus (HSV) transcription activator VP16. Monitored via elicited plant responses and in yeast reporter assays, TAL effector activity was inhibited without AD, while it was partly restored by the heterologous VP16-AD [23], [24], [25], [26].

Construction of TAL proteins with ordered repeats is challenging due to the highly repetitive nature of the repeats. We developed a modular cloning strategy to easily assemble TAL proteins and tested the TAL-dependent modulation of gene expression in human cells. We show that native TAL proteins function as transcriptional activators in different human cell lines and that this activity can be significantly enhanced by substituting the endogenous AD by the VP16 AD. Importantly, specifically designed TAL effector derivatives were shown to induce the expression of an exogenous reporter gene as well as of target endogenous genes.

## Results

### The TAL protein-AD can be functionally replaced by ADs from other organisms

**Figure 1:**
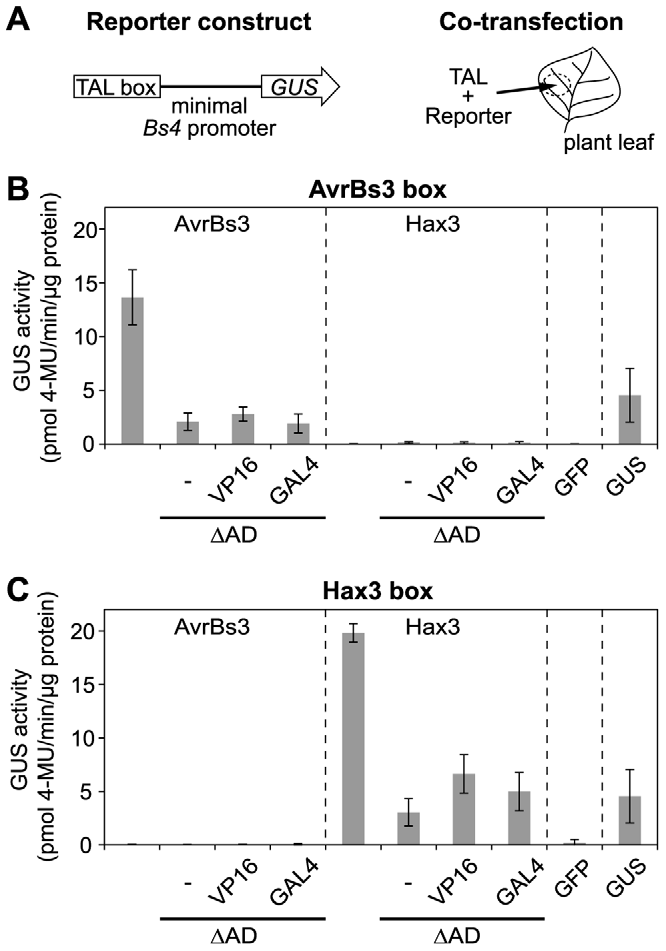
The VP16 or GAL4 activation domains (ADs) can partially complement the endogenous ADs of AvrBs3 and Hax3 *in planta*. (A) Schematic drawing of reporter construct and co-transfection experiment. Target AvrBs3 and Hax3 boxes were inserted upstream of the minimal *Bs4* promoter into a GUS reporter vector [7]. GUS reporter and constructs driving expression of *TAL* genes were co-delivered via *Agrobacterium* into *N. benthamiana* leaf cells. (B) Specific induction of the AvrBs3 box by AvrBs3, an AD deletion mutant (ΔAD), the ΔAD mutant complemented with the VP16-AD (VP16), and the ΔAD mutant complemented with the GAL4-AD (GAL4). (C) Specific induction of the Hax3 box by Hax3 and AD-derivatives. (B, C) GFP and constitutively expressed GUS served as negative and positive controls, respectively. Samples were taken 2 dpi (error bars indicate SD; n = 3). GUS activity was measured with MUG (4-methylumbelliferyl-β-D-glucuronide) as substrate. 4-MU, 4-methylumbelliferone.

To generate TAL proteins with a potential transcription factor activity in non-plant organisms, we deleted the ADs from the TAL effectors AvrBs3 and Hax3 and replaced them with the ADs of the HSV VP16 and the yeast GAL4 transcription activators, respectively (Figure S1A). All TAL proteins were designed such that they contained an N-terminal tag of green fluorescent protein (GFP). Following heterologous expression, all TAL proteins were confirmed to localize to plant nuclei indicating functionality of their nuclear localization signals (NLSs) and thus protein integrity (Figure S2). To determine the transcription factor activity of the TAL effector-derivatives in plant cells, a reporter assay was set up. Target DNA sequences specifically recognized by AvrBs3 and Hax3 (termed AvrBs3- and Hax3-box, respectively) were inserted upstream of a minimal promoter which has low background activity [27] into a reporter vector containing a promoterless *uidA* (β-glucuronidase, GUS) reporter gene [7] (Figures 1A and S1B). The reporter constructs were co-transfected into *Nicotiana benthamiana* leaf cells together with constructs that mediated the expression of the *avrBs3-* and *hax3*-derivatives, respectively (Figure 1A).

Quantification of GUS activity revealed that AvrBs3 and Hax3 only induced promoters that contained the AvrBs3- and Hax3-box, respectively. As expected, deletion of the ADs strongly reduced the transcription factor activity of both effectors (Figure 1B,C). Addition of the VP16-AD and GAL4-AD led to a slight increase of the AvrBs3- and Hax3-activities *in planta*; however, activity was still substantially lower than that of the original TAL proteins (Figure 1B,C). These data show that AvrBs3 and Hax3 still contain approx. 15% of their activity after deletion of the AD, and that the VP16-AD only partially complements the natural TAL protein-AD *in planta*.

### TAL protein-directed transcriptional activation in human cells

Plant-infecting xanthomonads are not pathogenic to humans and there is no indication that the bacteria can translocate TAL effectors into cells of non-plant organisms. TAL proteins were nevertheless expected to specifically interact and to even function with matching DNA-sequences in non-plant organisms. This notion was fuelled by the observation that *in planta* as well as *in vitro,* the specificity of TAL protein-DNA interactions is solely dependent on the TAL protein repeat composition [5], [6], [10], [28]. Moreover, there are no indications for the existence of kingdom specific eukaryotic host factors contributing to TAL protein functions.

**Figure 2:**
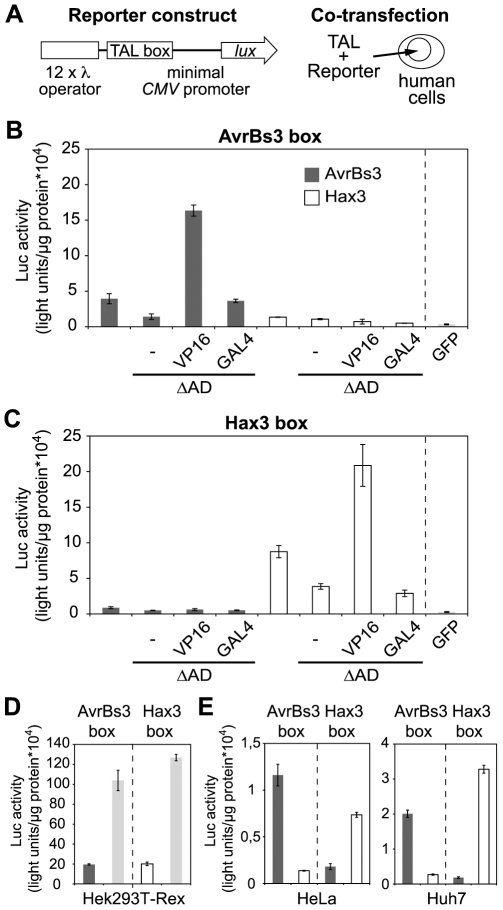
AvrBs3 and Hax3 activate transcription in human cells. (A) Schematic drawing of reporter construct and co-transfection experiment. TAL boxes were cloned upstream of a minimal cytomegalovirus (CMV) promoter into a luciferase (*luc*) reporter vector. The λ operators are bound by a chimeric transactivator used as control. The Luc reporter plasmids were co-transfected into human Hek293T-Rex cells together with expression plasmids where transcription of the *TAL* or *GFP* genes was driven by a fully active *CMV* promoter. (B) Specific induction of the AvrBs3 box by AvrBs3, an AD deletion mutant (ΔAD), the ΔAD mutant complemented with the VP16-AD (VP16), and the ΔAD mutant complemented with the GAL4-AD (GAL4). (C) Specific induction of the Hax3 box by Hax3 and AD-derivatives. (D) Comparison of induction levels of the Luc reporter plasmids using Hax3 with VP16-AD or via co-transfection of a plasmid expressing a chimeric transactivator for the λ operator and addition of the inducer coumermycin (light grey bars), respectively, in Hek293T-Rex cells. (E) Specific activity of AvrBs3 and Hax3 containing the VP16-AD, respectively, in human Huh7, and HeLa cells. (B-E) Measurements were done 1 dpi. Luc activity was plotted in arbitrary light units per µg protein. Dark grey bars: AvrBs3, white bars: Hax3. The activation is statistically significant (relative to GFP) with P≤0.005 for all values except AvrBs3-ΔAD, P≤0.01.

To test if TAL proteins were able to direct gene expression in human cells, we established reporter constructs that contained the AvrBs3- or Hax3-box upstream of a minimal human cytomegalovirus (CMV) promoter and a luciferase (*luc*) reporter gene (Figures 2A and S1B). When either reporter plasmid was co-transfected into human Hek293T-Rex cells together with a plasmid that expressed GFP via a fully active CMV promoter, only weak luciferase activity was detectable. This confirmed that the minimal CMV promoter only has a low basal activity (Figure 2B,C). In contrast, co-expression of native AvrBs3 or Hax3 resulted in a specific activation of promoters with matching target DNA boxes, respectively (Figure 2B,C). Interestingly, the transcriptional activation was low when we applied AvrBs3- and Hax3-derivatives with deleted ADs. Conversely, AvrBs3 and Hax3 that contained a VP16-AD caused a strong transcriptional activation of the luciferase reporter gene, i.e., transcription activation was 2–3 times higher than that induced by the original (*in planta* active) AvrBs3 and Hax3 and 50–80 times higher than transcription in the negative control (Figure 2B,C). In comparison, the GAL4-AD did not increase the activity of the ΔAD-TAL proteins significantly. Importantly, all expressed TAL proteins were found to localize to the nucleus of the human cells indicating correct nuclear import in the heterologous host (Figure S3A). Protein levels of TAL effector-derivatives expressed in human cells were comparable (Figure S3B,C). Our data show that TAL proteins are capable to specifically induce gene expression in human cells and that substitution of the endogenous AD by a VP16-AD strongly increases transcriptional activity.

Next, we analyzed whether the TAL protein-directed transcriptional activation was functional in different human cell lines. As also shown in Figure 2D, analogous experiments that were performed with TAL-VP16 derivatives and reporter constructs in Hek293T-Rex (human embryonic kidney) cells, Huh7 (hepatocarcinoma) cells, and HeLa (cervical cancer) cells led in each case to an evident and specific induction of reporter gene activity in matching TAL protein-target box combinations (Figure 2D). In sum, these results demonstrate that TAL effector-derivatives are functional as transcriptional activators in different human cells.

### A modular cloning strategy to assemble TAL proteins with designed repeat architecture

**Figure 3:**
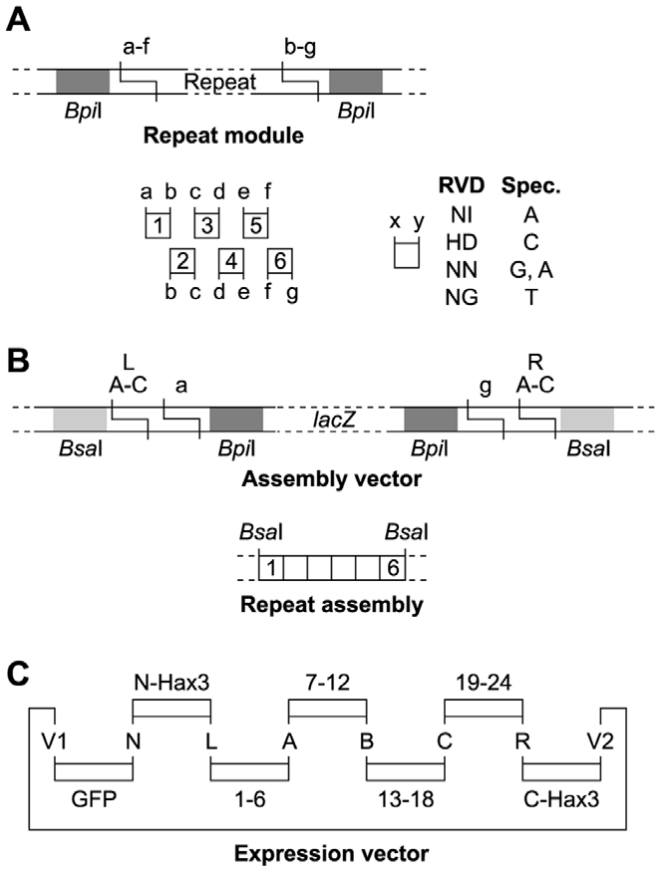
Golden TAL Technology for assembly of TAL proteins with programmed repeat composition. (A) Single TAL repeats were cloned with flanking *Bpi*I sites that generate specific four base pair-overhangs (a–g). Matching sites are indicated by identical letters. A library was constructed with four different repeat types (RVD, repeat variable di-residue: NI, HD, NN, NG) for each repeat position. The repeat types have different DNA-specificities (Spec., only upper DNA-strand is shown). (B) One to six repeats are assembled in specific order into an assembly vector to generate a repeat assembly with flanking *Bsa*I sites. (C) TALs were directly assembled with N-terminal GFP-tag into an expression vector using fragments with matching *Bsa*I-generated overhangs (capital letters). Insertion of one to four repeat assemblies generated TALs with 1 to 24 repeats. The last repeat is only a half repeat as typical for TAL proteins. Please see the Text S1 and Figure S4 for details.

To target defined DNA sequences and individual promoters with TAL proteins it is required to control their order of repeats. Unfortunately, the repetitive nature of TAL effector repeats makes it inherently complicated to use classical or PCR-based cloning techniques to generate TAL proteins with a given order of repeats. For that reason, we established a rapid and flexible cloning technique (Figure 3 and S4, see Text S1 for details).

Single TAL protein repeats were cloned as individual modules with flanking *Bpi*I restriction sites. The type IIs sites were designed to generate specific overhangs that determine the position of the repeat in a repeat array (Figure 3A) while preserving the amino acid sequence. For each repeat position, four key repeats (NI, HD, NN, NG) were cloned that specify the four DNA base pairs. Using a *Bpi*I cut-ligation reaction, one to six repeats can be combined into an assembly vector that contained additional flanking *Bsa*I sites which specify the position of the repeat assembly in the final repeat domain (Figure 3B). In addition, *Bsa*I-flanked modules of the TAL protein N-terminal region and C-terminal region fused to VP16, and an N-terminal GFP-module were designed. In a *Bsa*I cut-ligation reaction, these modules can be specifically aligned into a vector backbone (Figure 3C). With a plasmid library of only 29 repeat modules and seven different assembly vectors, synthetic TAL proteins with 1 to 24 specific repeats can be generated in a matter of days. The modular technology also allows a simple integration of an expanded module toolbox with different TAL protein termini, tags, and vectors. Since this toolbox is essentially based on golden gate cloning [29], [30], we termed this cloning strategy “Golden TAL Technology”.

### Designed TAL proteins direct the expression of endogenous human genes

Next, we considered it important to evaluate whether synthetic TAL proteins were also applicable to activate the transcription and thus the expression of different endogenous human genes. Accordingly, we applied the Golden TAL Technology to generate artificial TAL proteins with repeats that matched DNA sequences in promoter regions of the human *PUMA* (p53 upregulated modulator of apoptosis), an interferon alpha (*IFNa1*), and an interferon beta (*IFNβ1*) gene, respectively (Figure S5A). PUMA is a pro-apoptotic member of the Bcl-2 protein family [31], [32], whereas IFNα1 and IFNβ1belong to type I interferons that are involved in the innate immune response in humans against viral infections [33], [34]. To efficiently direct human gene expression, the designed “human TAL proteins” (HuTALs) contained a C-terminal VP16 activation domain. Protein levels of HuTALs expressed in human cells were comparable and the proteins localized to the nucleus of human cells (Figure S5B,C).

**Figure 4:**
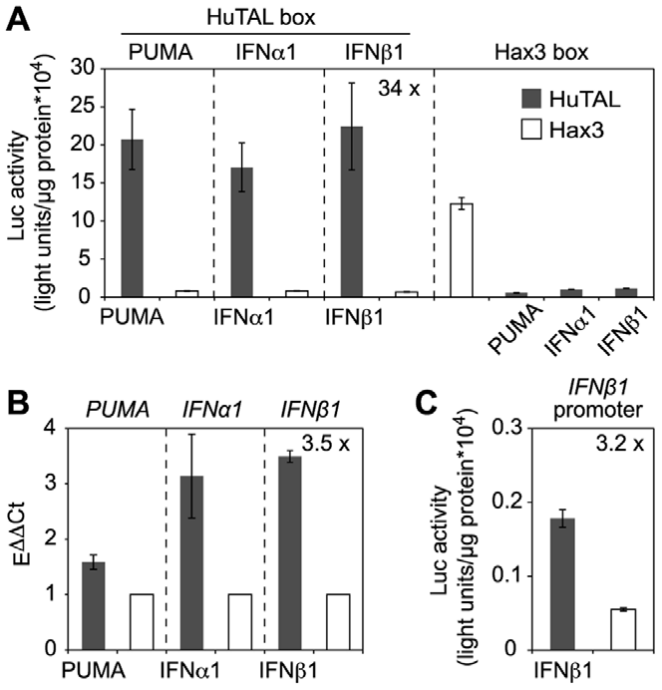
Designed TAL proteins induce expression of human genes. Artificial TAL proteins (HuTALs) containing a VP16-AD were constructed that target promoter regions of human *PUMA*, *IFNα1*, or *IFNβ1* genes. (A) Target DNA boxes were cloned upstream of the minimal CMV promoter into the *luc* reporter vector. Luc reporter constructs were co-transfected with constructs driving *TAL* gene expression into human Hek293T-Rex cells. Measurements were done 1 dpi (error bars indicate SD). Luc activity was plotted in arbitrary light units per µg protein. (B) TAL protein-directed expression of endogenous human genes. Plasmids expressing HuTALs or Hax3 were transfected into human Hek293T-Rex cells. mRNAs levels of target genes were determined by quantitative real-time PCR. mRNA levels in presence of Hax3 (negative control, white bars) were set to 1. (C) The natural *IFNβ1* promoter was cloned into the luc reporter vector. The IFNβ1 reporter was co-transfected with constructs directing expression of *HuTAL_IFN_β_1_* or *hax3*, respectively. Dark grey bars: HuTAL, white bars: Hax3. (A,C) P≤0.005 for all values; (B) *PUMA*, P = 0.0051; *IFNα1*, P = 0.11; *IFNβ1*, P = 0.00028.

As an important pilot experiment, we first inserted the predicted target DNA boxes of the HuTALs into the reporter vector described above, i.e., upstream of the minimal CMV promoter and the promoterless *luc* gene (Figure 2A). Co-transfection of human Hek293T-Rex cells with the reporter constructs and *HuTAL* expression plasmids resulted in a strong (22- to 34-fold) and specific induction of reporter activity only in matching TAL protein-target DNA box combinations (Figure 4A). Thus, we confirmed the specificity and functionality of the HuTALs.

Next, we transfected Hek293T-Rex cells with the CMV promoter-driven *HuTAL* genes to address if we could also affect the expression of endogenous human genes by the HuTALs. These experiments revealed that expression of the respective HuTAL in each case caused moderately increased levels of target endogenous human gene transcript levels (Figure 4B). While *PUMA* transcript levels were only slightly induced (1.5 x), the *IFNα1* and *IFNβ1* levels were 3.1-fold and 3.5-fold up-regulated, respectively (Figure 4B).

TAL protein-directed induction of endogenous gene expression was considerably lower than with our reporter constructs. This prompted us to determine if the promoter *per se* has an influence on TAL protein functionality. Hence, we replaced the minimal CMV promoter in the reporter plasmid by the native *IFNβ1* promoter sequence. Co-transfection of the *IFNβ1* reporter construct with the HuTAL targeting this promoter led to a moderate, 3.2-fold induction of reporter activity (Fig. 4C) comparable to the level of endogenous gene induction. This suggests that the efficiency of TAL proteins also depends on the individual target promoter. Taken together, our experiments show that TAL protein-derivatives can be used to specifically induce expression of human endogenous genes and demonstrate the functionality of the TAL protein technology in other organisms besides plants.

## Discussion

In this report we demonstrate that TAL protein derivatives can be used to specifically modulate the transcription of genes in human cells. Our findings confirm and extend the recent work of Miller *et al.* [14] and Zhang *et al.* [17]. In addition to those studies, we report here that native *Xanthomonas* TAL effectors (AvrBs3 and Hax3) exhibited considerable activity in the heterologous host cells. This is surprising considering that these are proteins from bacterial origin that were evolutionary shaped to function in plant cells. Two scenarios are conceivable to interpret this finding. On one hand, the mechanism that is applied by TAL proteins to activate the host's transcription machinery may be based on the protein structure itself. Alternatively, TAL proteins may rely on elements that are conserved in the different hosts. Hence, it will be interesting to see if TAL proteins function ubiquitously in all eukaryotes or if there is a specific host determinant required for activity.

Deletion of the endogenous TAL protein activation domain (AD) strongly compromized transcriptional activation, but a measurable residual activity remained (Figs. 1 and 2). This finding supports two conclusions. First, the endogenous TAL-AD is clearly a major determinant of TAL protein function also in heterologous hosts, and, second, TAL proteins have a low but specific residual transcriptional activation activity, irrespective of the presence of the AD. This has not been demonstrated before, and suggests that this low activity is based on TAL-DNA interactions, which are not influenced by the AD [5], or possible AD-independent interactions with components of the host transcription machinery.

The substitution of the endogenous AD by the heterologous HSV VP16-AD strongly elevated the activity of AvrBs3 and Hax3 ΔAD derivatives in human cells but not in plant cells (Figs. 1, 2 and 4). The human virus AD is adapted to function in human cells, and this function can be transferred to TAL proteins. Miller *et al*. [14] and Zhang *et al.* [17] applied artificial TAL proteins, where the VP16-AD was fused to full-length or C-terminally truncated TAL proteins. The TAL proteins designed by Miller *et al.* [14] and Zhang *et al.* [17] as well as our synthetic TAL proteins each activated gene expression in human cells indicating that the VP16-AD functions in two ways, as a substitute or as a supplement of the endogenous TAL-AD. In addition, we showed that TAL-VP16 fusions are active in different human cell lines (Fig. 2D). The TAL protein activity strongly varied between HEK cells showing highest and HeLa cells showing lowest transcriptional activation, respectively. Likely, this is in part due to different transfection efficiencies that were achieved with the different cell lines. Further studies will clarify if tissue-specific requirements exist or whether different TAL protein backbones (N- and C-termini) function differently well in human cells.

We designed the Golden TAL Technology as a toolbox for a flexible assembly of TAL proteins with designed repeats, different N-, C-termini, and tags. While our method is somewhat similar to the recently published method of Zhang *et al*. [17], it includes a number of aspects that we consider advantegous for the construction of designer TAL proteins. First, our procedure does not rely on PCR but instead uses plasmid sub-clones of single repeats which is user-friendly and prohibits PCR-based errors. Furthermore, Zhang *et al*. [17] altered the codon usage between TAL protein repeats to allow efficient PCR, while we preferred to keep the codon usage near-identical between repeats to reduce repeat-specific effects on translation. Second, our library consists of only 24 single-repeat plasmids, instead of the 48 PCR-library of Zhang *et al*. [17]. Third, our method allows construction of TAL proteins with a flexible number of repeats. At present TAL proteins with 1 to 24 repeats can be constructed based on assembly modules containing six or less individual repeats. Fourth, we designed the restriction overhangs such that they differ each at a minimum of two positions. We found this to be essential for an efficient error-free ligation. Finally, we employ the full power of Golden Gate cloning which is a fast and versatile method to assemble many fragments specifically in a one-step cut-ligation reaction [29], [30]. To acknowledge this, we termed our cloning strategy Golden TAL Technology.

Taking advantage of the Golden TAL Technology we were able to straightforwardly construct artificial TAL proteins with VP16-AD and repeats that match to promoters of various human target genes. These tailored TAL proteins (HuTALs) strongly induced reporter gene activation more than 30-fold and also endogenous human *PUMA*, *IFNα1*, and *IFNβ1* mRNA levels. Endogenous gene induction was considerably weaker (1.5-fold to 3.5-fold), a phenomenon which was also observed with artificial ZF transcription factors [35], [36]. The efficiency of TAL protein-dependent endogenous human gene induction in the two recent studies varied. While Miller *et al*. [14] activated endogenous *NTF3* expression 20-fold, Zhang *et al*. [17] induced endogenous human *SOX2* and *KLF4* genes only 2.2 and 5.5 fold, respectively, or were not able to change expression levels of *c-MYC* and *OCT4* at all. This observation makes sense considering that in the transient transfection experiments, multiple reporter gene copies are applied, whereas the endogenous genes are present at much lower copy-number. Moreover, the epigenetic environment of the endogenous genes (nucleosome packaging, transcriptional interference with other regulatory factors, etc.) may significantly affect the activity of HuTALs. This may also explain, why in the case of the *PUMA* gene no phenotype (accelerated cell death) could be observed. Hence, future optimization of the HuTAL system will be necessary to allow efficient regulation of endogenous human gene expression.

A growing collection of studies shows that the programmable DNA-binding specificity of TAL proteins renders these proteins similarly useful as the ZF technology [19] for different biotechnology applications. Our work and the recently published ones [14], [17] are first crucial kick-off steps to use TAL proteins as highly potent and specific gene switches in human cells.

## Materials and Methods

### Plant growth and inoculations

*Nicotiana benthamiana* plants were cultivated in the greenhouse with day and night temperatures of 23°C and 19°C, respectively, 16 h light, and 40 to 60% humidity. Leaves of five- to seven-week-old plants were inoculated with an *Agrobacterium* suspension using a needleless syringe [7]. Inoculated plants were transferred to a Percival growth chamber (Percival Scientific) with day and night temperatures of 22°C and 18°C, respectively, and 16 h light.

### Cell culture and transfection

Hek293T-Rex, HeLa and Huh7 cells were grown under standard conditions [37] in DMEM (Invitrogen) supplemented with 10% FCS (Invitrogen), 1% non-essential amino acids and 1% penicillin/streptomycin (Invitrogen). Plasmids were transfected with Turbofect *in vitro* Transfection Reagent (Fermentas).

### Plasmids

For ΔAD constructs, the last 31 amino acids (aa) of AvrBs3 and Hax3 were deleted, respectively. 68 aa of VP16, or 113 aa of GAL4 were added as heterologous activation domains. Constructs for *TAL* expression *in planta* and in human cells were based on pVS300F [38] and pcDNA5 (Invitrogen), respectively. Reporter plasmids for plant cells were constructed as described [7]. Reporter plasmids for human cells were based on pF12A RM Flexi (Promega). The barnase gene of pF12A RM Flexi was replaced by a luciferase gene. To generate plasmids containing the recognition site of TALs, pF12A RM (Luc) was amplified with primers containing target boxes. The promoter and 5′ untranslated region (675 bp) of the *IFNβ1* promoter were amplified by PCR and inserted into the luciferase reporter plasmid. Further details on constructs and methods are given in the supplementary information and upon request.

### GUS assays

GUS assays from plant samples were essentially done as described [7]. Briefly, *Agrobacterium* strains delivering TAL effector constructs and GUS reporter constructs were mixed in equal amounts, and inoculated into *Nicotiana benthamiana* leaves with an OD_600_ of 0.8. Two days post infiltration (dpi), two leaf discs (0.9 cm diameter) were sampled and quantitative GUS activity was determined using 4-methyl-umbelliferyl-β-D-glucuronide (MUG). Proteins were quantified by Bradford assays (BioRad). Data points correspond to triplicate samples from different plants.

### qRT-PCR, western blotting and luciferase assay

Human cells were harvested 24 h after transfection with plasmids. For RNA extraction, a standard protocol was used as described [37]. RNA was heated for 5 min to 70°C and reverse transcription of DNaseI (Roche), *Eco*RI, and *Bgl*II (Fermentas) treated RNA (2 µg) was performed by RevertAid H Minus Reverse Transcriptase (Fermentas) for 1 h at 42°C, followed by heat inactivation for 10 min at 70°C as described in the manufacturer's manual. Quantitative PCRs (for primers see Table S1) were performed with the QuantiTect SYBR Green PCR Kit (Qiagen) according to the manufacturer's manual using a LightCycler 2.0 instrument (Roche). PCR conditions were: 95°C 15 min, (95°C, 10 sec; 60°C, 25 sec; 72°C 25 sec) x 45 cycles. The qRT-PCR data were normalized to GAPDH as internal control and analyzed by the ddCt method [39]. For western blotting total protein extracts were prepared with Luciferase Cell Culture Lysis Reagent (Promega) and analyzed by western blotting for GFP (α-GFP, Invitrogen) and Vinculin (hVin-1, Sigma-Aldrich). Luciferase assays were prepared as described in the Luciferase Assay System (Promega). The data points were generated from three biological replicates (separate transfections).

### Golden TAL Technology

For details on individual modules, vectors, and cloning strategy see Text S1 and Figure S4. Briefly, single repeats were PCR-amplified with flanking *Bpi*I sites (for primers see Table S1) and subcloned by cut-ligation using *Sma*I and T4-DNA ligase into a pUC57-derivative (Amp) with mutated *Bsa*I site. The assembly vectors (Kan) contain a *lacZ* gene flanked by *Bpi*I sites which is replaced in cloning step 1 by the inserted repeats and used for blue-white selection of correct assembly products. *Bsa*I-flanked modules are assembled in cloning step 2 into a target expression vector. Assembly reactions were set up as cut-ligation with 50-100 ng of each plasmid module, 1 µl *Bsa*I (NEB), 2 µl ATP (10 mM), 2 µl restriction buffer no. 4 (NEB), 1 µl T4-DNA ligase (5 u/µl) in a 20 µl reaction for 1 h at 37°C followed by 20 min. inactivation of enzymes at 70°C.

## Supporting Information

Text S1 TAL gene expression constructs, reporter constructs, the Golden TAL Technology toolbox, and promoter regions of human genes chosen as targets for TAL protein-directed expression.
(DOC)

Figure S1 TAL effector-derivatives and reporter constructs. (*A*) Functional domains of TAL effector-derivatives used. The endogenous C-terminal transcriptional activation domain (AD; C-terminal 31 aa) was deleted or replaced by the ADs from herpes simplex virus VP16 (C-terminal 68 aa) or yeast GAL4 (C-terminal 113 aa). NLS: nuclear localization signals; red: repeat domain of AvrBs3 and derivatives; yellow: repeat domain of Hax3 and derivatives. All constructs carried an N-terminal green fluorescent protein (GFP) tag. (*B*) Reporter constructs used for transient expression in plant and human cells, respectively. For experiments in plants, TAL protein target DNA boxes were inserted in front of a minimal *Bs4* promoter which has low basal activity upstream of a promoterless *uidA* (GUS) reporter gene as described [4]. For studies with human cells, TAL protein target DNA boxes were placed upstream of the minimal *CMV* promoter and a promoterless luciferase (*luc*) gene into a pF12A RM Flexi (Promega) reporter vector. To study activity of the endogenous interferon β promoter, the minimal *CMV* promoter was replaced by part of the *INFβ* promoter.
(TIF)

Figure S2 GFP-TAL protein fusions localized to the plant nucleus. GFP-TAL protein fusions or GFP alone were expressed transiently in leaf cells of *Nicotiana benthamiana* via *Agrobacterium*-mediated delivery. Two days post infiltration, *N. benthamiana* leaf epidermis cells were stained with DAPI (4′,6-diamidino-2-phenylindole) and analyzed by confocal laser scanning microscopy. Green: GFP fluorescence; blue: DAPI fluorescence; red: chlorophyll autofluorescence. Scale bars indicate 50 µm.
(TIF)

Figure S3 GFP-TAL protein fusions are expressed and localize to the nucleus in human cells. (*A*) GFP-TAL protein fusions or GFP alone were expressed after transfection of human Hek293T-Rex cells. One day post transfection, cells were stained with DAPI (4′,6-diamidino-2-phenylindole) and analyzed by fluorescence microscopy. Green: GFP fluorescence; blue: DAPI fluorescence. (*B*) Western-blot analysis of whole cell extracts of transfected Hek293T-Rex cells. (*C*) TAL protein-derivatives expressed in HeLa cells show degradation patterns. (*B*+*C*) GFP and GFP-TAL protein fusions were detected using α-GFP antibody. An α-Vinculin antibody was used as constitutive loading control.
(TIF)

Figure S4 Golden TAL Technology toolbox. Single repeat modules were subcloned with flanking *Bpi*I sites that specify their position in the repeat array. A library of four repeat types (NI = A, HD = C, NN = G/A, NG = T) was constructed for each of the six repeat positions. Stop repeats (1s to 5s) can be used to terminate the repeat assembly. In cloning step 1, up to six repeats are inserted into an assembly vector to generate a repeat assembly. Different assembly vectors are used to position the repeat assembly within the final TAL protein repeat domain. In cloning step 2, individual repeat assemblies, Hax3 N- and C-termini, and an N-terminal GFP-tag are ligated into an expression vector to generate the complete TAL gene.
(TIF)

Figure S5 HuTALs designed to target expression of human genes. (*A*) HuTALs with 17.5 to 19.5 repeats matching a DNA sequence (box) in the promoter region of target genes are assembled as N-terminal GFP fusions using the “Golden TAL technology”. (*B*) GFP-TAL protein fusions were expressed after transfection of human Hek293T-Rex cells. One day post transfection, cells were stained with DAPI (4′,6-diamidino-2-phenylindole) and analyzed by fluorescence microscopy. Green: GFP fluorescence; blue: DAPI fluorescence. (*C*) Western-blot analysis of whole cell extracts of Hek293T-Rex cells transfected with HuTAL expression constructs.
(TIF)

Table S1 Selected primers used in this study.
(DOC)
