# Supplementary material for: Transcriptional Activators of Human Genes with Programmable DNA-Specificity
Source: PLoS One. 2011 May 19;6(5):e19509. doi: 10.1371/journal.pone.0019509 (PMC3098229; doi:10.1371/journal.pone.0019509)
Supplement: Text S1 — TAL gene expression constructs, reporter constructs, the Golden TAL Technology toolbox, and promoter regions of human genes chosen as targets for TAL protein-directed expression. (DOC) [file pone.0019509.s001.doc]

**Supporting Information - Text S1**

***TAL* expression construct**

**GFP-Hax3-ΔAD-VP16**

The AvrBs3- and Hax3-derivatives were constructed using classical restriction enzymes and transferred into expression vectors as *Eco*RI-*Xho*I fragment. As an example, the final sequence of the Hax3-derivative carrying the VP16-AD is shown. The N-terminal GFP is highlighted in green; Hax3 ΔAD is in bold letters; the VP16 AD is highlighted in yellow; restriction sites used for cloning are indicated or underlined.

*Eco*RI

ATGGTGAGCAAGGGCGAGGAGCTGTTCACCGGGGTGGTGCCCATCCTGGTCGAGCTGGACGGCGACGTAAACGGCCACAAGTTCAGCGTGTCCGGCGAGGGCGAGGGCGATGCCACCTACGGCAAGCTGACCCTGAAGTTCATCTGCACCACCGGCAAGCTGCCCGTGCCCTGGCCCACCCTCGTGACCACCCTGACCTACGGCGTGCAGTGCTTCAGCCGCTACCCCGACCACATGAAGCAGCACGACTTCTTCAAGTCCGCCATGCCCGAAGGCTACGTCCAGGAGCGCACCATCTTCTTCAAGGACGACGGCAACTACAAGACCCGCGCCGAGGTGAAGTTCGAGGGCGACACCCTGGTGAACCGCATCGAGCTGAAGGGCATCGACTTCAAGGAGGACGGCAACATCCTGGGGCACAAGCTGGAGTACAACTACAACAGCCACAACGTCTATATCATGGCCGACAAGCAGAAGAACGGCATCAAGGTGAACTTCAAGATCCGCCACAACATCGAGGACGGCAGCGTGCAGCTCGCCGACCACTACCAGCAGAACACCCCCATCGGCGACGGCCCCGTGCTGCTGCCCGACAACCACTACCTGAGCACCCAGTCCGCCCTGAGCAAAGACCCCAACGAGAAGCGCGATCACATGGTCCTGCTGGAGTTCGTGACCGCCGCCGGGATCACTCTCGGCATGGACGAGCTGTACAAGATCGAT**ATGGATCCCATTCGTTCGCGCACACCAAGTCCTGCCCGCGAGCTTCTGTCCGGACCCCAACCCGATGGGGTTCAGCCGACTGCAGATCGTGGGGTGTCTCCGCCTGCCGGCGGCCCCCTGGATGGCTTGCCCGCTCGGCGGACGATGTCCCGGACCCGGCTGCCATCTCCCCCTGCCCCCTCACCTGCGTTCTCGGCGGACAGCTTCAGTGACCTGTTACGTCAGTTCGATCCGTCACTTTTTAATACATCGCTTTTTGATTCATTGCCTCCCTTCGGCGCTCACCATACAGAGGCTGCCACAGGCGAGTGGGATGAGGTGCAATCGGGTCTGCGGGCAGCCGACGCCCCCCCACCCACCATGCGCGTGGCTGTCACTGCCGCGCGGCCGCCGCGCGCCAAGCCGGCGCCGCGACGACGTGCTGCGCAACCCTCCGACGCTTCGCCGGCCGCGCAGGTGGATCTACGCACGCTCGGCTACAGCCAGCAGCAACAGGAGAAGATCAAACCGAAGGTTCGTTCGACAGTGGCGCAGCACCACGAGGCACTGGTCGGCCATGGGTTTACACACGCGCACATCGTTGCGCTCAGCCAACACCCGGCAGCGTTAGGGACCGTCGCTGTCAAGTATCAGGACATGATCGCAGCGTTGCCAGAGGCGACACACGAAGCGATCGTTGGCGTCGGCAAACAGTGGTCCGGCGCACGCGCTCTGGAGGCCTTGCTCACGGTGGCGGGAGAGTTGAGAGGTCCACCGTTACAGTTGGACACAGGCCAACTTCTCAAGATTGCAAAGCGTGGCGGCGTGACCGCAGTGGAGGCAGTGCATGCATGGCGCAATGCACTGACGGGTGCCCCCCTGAACCTGACCCCGGAGCAGGTGGTGGCCATCGCCAGCAATATTGGTGGCAAGCAGGCGCTGGAGACGGTGCAGCGGCTGTTGCCGGTGCTGTGCCAGGCCCATGGCCTGACCCCGCAGCAGGTGGTGGCCATCGCCAGCCACGATGGCGGCAAGCAGGCGCTGGAGACGGTGCAGCGGCTGTTGCCGGTGCTGTGCCAGGCCCATGGCCTGACCCCGGAGCAGGTGGTGGCCATCGCCAGCAATATTGGTGGCAAGCAGGCGCTGGAGACGGTGCAGGCGCTGTTGCCGGTGCTGTGCCAGGCCCATGGCCTGACCCCGGAGCAGGTGGTGGCCATCGCCAGCCACGATGGCGGCAAGCAGGCGCTGGAGACGGTGCAGCGGCTGTTGCCGGTGCTGTGCCAGGCCCATGGCCTGACCCCGCAGCAGGTGGTGGCCATCGCCAGCCACGATGGCGGCAAGCAGGCGCTGGAGACGGTGCAGCGGCTGTTGCCGGTGCTGTGCCAGGCCCATGGCCTGACCCCGCAGCAGGTGGTGGCCATCGCCAGCCACGATGGCGGCAAGCAGGCGCTGGAGACGGTGCAGCGGCTGTTGCCGGTGCTGTGCCAGGCCCATGGCCTGACCCCGCAGCAGGTGGTGGCCATCGCCAGCAATAGCGGTGGCAAGCAGGCGCTGGAGACGGTGCAGCGGCTGTTGCCGGTGCTGTGCCAGGCCCATGGCCTGACCCCGCAGCAGGTGGTGGCCATCGCCAGCAATAGCGGTGGCAAGCAGGCGCTGGAGACGGTGCAGCGGCTGTTGCCGGTGCTGTGCCAGGCCCATGGCCTGACCCCGCAGCAGGTGGTGGCCATCGCCAGCAATAGCGGTGGCAAGCAGGCGCTGGAGACGGTGCAGCGGCTGTTGCCGGTGCTGTGCCAGGCCCATGGCCTGACCCCGGAGCAGGTGGTGGCCATCGCCAGCCACGATGGCGGCAAGCAGGCGCTGGAGACGGTGCAGCGGCTGTTGCCGGTGCTGTGCCAGGCCCATGGCCTGACCCCGGAGCAGGTGGTGGCCATCGCCAGCAATATTGGTGGCAAGCAGGCGCTGGAGACGGTGCAGCGGCTGTTGCCGGTGCTGTGCCAGGCCCATGGCCTGACCCCGCAGCAGGTGGTGGCCATCGCCAGCAATGGCGGCGGCAGGCCGGCGCTGGAGAGCATTGTTGCCCAGTTATCTCGCCCTGATCCGGCGTTGGCCGCGTTGACCAACGACCACCTCGTCGCCTTGGCCTGCCTCGGCGGACGTCCTGCGCTGGATGCAGTGAAAAAGGGATTGCCGCACGCGCCGGCCTTGATCAAAAGAACCAATCGCCGTATTCCCGAACGCACATCCCATCGCGTTGCCGACCACGCGCAAGTGGTTCGCGTGCTGGGTTTTTTCCAGTGCCACTCCCACCCAGCGCAAGCATTTGATGACGCCATGACGCAGTTCGGGATGAGCAGGCACGGGTTGTTACAGCTCTTTCGCAGAGTGGGCGTCACCGAACTCGAAGCCCGCAGTGGAACGCTCCCCCCAGCCTCGCAGCGTTGGGACCGTATCCTCCAGGCATCAGGGATGAAAAGGGCCAAACCGTCCCCTACTTCAACTCAAACGCCGGATCAGGCGTCTTTGCATGCATTCGCCGATTCGCTGGAGCGTGACCTTGATGCGCCTAGCCCAATGCACGAGGGAGATCAGACGCGGGCAAGCAGCCGTAAACGGTCCCGATCGGATCGTGCTGTCACCGGTCCCTCCGCACAGCAATCGTTCGAGGTGCGCGTTCCCGAACAGCGCGATGCGTTGCATTTGCCCCTCCTCAGCTGGGGTGTAAAACGCCCGCGTACCAGGATCGGCGGCCTCCTGGATCCTGGTACGCCCATGGATGCCGACCTGGTAGCGTCCAGCACCGTGGTTTGG**TCTAGAGAGCTCCACTTAGACGGCGAGGACGTGGCGATGGCGCATGCCGACGCGCTAGACGATTTCGATCTGGACATGTTGGGGGACGGGGATTCCCCGGGGCCGGGATTTACCCCCCACGACTCCGCCCCCTACGGCGCTCTGGATACGGCCGACTTCGAGTTTGAGCAGATGTTTACCGATGCCCTTGGAATTGACGAGTACGGTGGGTAG

*Hin*dIII-*Xho*I

**Reporter construct**

**Luciferase reporter construct to measure TAL protein activity in human cells.**

Partial nucleotide sequence of the Hax3 box reporter vector, a pF12A RM Flexi (Promega; GenBank: EF030520)-derivative containing a luciferase reporter gene. Uppercase letters: luciferase coding region; bold font: Hax3 box; yellow shading: minimal CMV promoter; blue shading: 12 x λ operator; underlined: restriction sites used for cloning. Reporter constructs for other TAL proteins carried the corresponding TAL box instead of the Hax3 box at the same position.

agtactccgctcgagtttacctctggcggtgatagtcgagtttacctctggcggtgatagtcgagtttacctctggcggtgatagtcgagtttacctctggcggtgatagtcgagtttacctctggcggtgatagtcgagtttacctctggcggtgatagtcgagtttacctctggcggtgatagtcgagtttacctctggcggtgatagtcgagtttacctctggcggtgatagtcgagtttacctctggcggtgatagtcgagtttacctctggcggtgatagtcgagtttacctctggcggtgatagtcgactctagaaagat**TACACCCAAACAT**taggcgtgtacggtgggaggcctatataagcagagctggtttagtgaaccgtcagatccctggagacgccatccacgctgttttgacctccatagaagacaccgggaccgatcaacctaagctttccATGGAAGACGCCAAAAACATAAAGAAAGGCCCGGCGCCATTCTATCCGCTGGAAGATGGAACCGCTGGAGAGCAACTGCATAAGGCTATGAAGAGATACGCCCTGGTTCCTGGAACAATTGCTTTTACAGATGCACATATCGAGGTGGACATCACTTACGCTGAGTACTTCGAAATGTCCGTTCGGTTGGCAGAAGCTATGAAACGATATGGGCTGAATACAAATCACAGAATCGTCGTATGCAGTGAAAACTCTCTTCAATTCTTTATGCCGGTGTTGGGCGCGTTATTTATCGGAGTTGCAGTTGCGCCCGCGAACGACATTTATAATGAACGTGAATTGCTCAACAGTATGGGCATTTCGCAGCCTACCGTGGTGTTCGTTTCCAAAAAGGGGTTGCAAAAAATTTTGAACGTGCAAAAAAAGCTCCCAATCATCCAAAAAATTATTATCATGGATTCTAAAACGGATTACCAGGGATTTCAGTCGATGTACACGTTCGTCACATCTCATCTACCTCCCGGTTTTAATGAATACGATTTTGTGCCAGAGTCCTTCGATAGGGACAAGACAATTGCACTGATCATGAACTCCTCTGGATCTACTGGTCTGCCTAAAGGTGTCGCTCTGCCTCATAGAACTGCCTGCGTGAGATTCTCGCATGCCAGAGATCCTATTTTTGGCAATCAAATCATTCCGGATACTGCGATTTTAAGTGTTGTTCCATTCCATCACGGTTTTGGAATGTTTACTACACTCGGATATTTGATATGTGGATTTCGAGTCGTCTTAATGTATAGATTTGAAGAAGAGCTGTTTCTGAGGAGCCTTCAGGATTACAAGATTCAAAGTGCGCTGCTGGTGCCAACCCTATTCTCCTTCTTCGCCAAAAGCACTCTGATTGACAAATACGATTTATCTAATTTACACGAAATTGCTTCTGGTGGCGCTCCCCTCTCTAAGGAAGTCGGGGAAGCGGTTGCCAAGAGGTTCCATCTGCCAGGTATCAGGCAAGGATATGGGCTCACTGAGACTACATCAGCTATTCTGATTACACCCGAGGGGGATGATAAACCGGGCGCGGTCGGTAAAGTTGTTCCATTTTTTGAAGCGAAGGTTGTGGATCTGGATACCGGGAAAACGCTGGGCGTTAATCAAAGAGGCGAACTGTGTGTGAGAGGTCCTATGATTATGTCCGGTTATGTAAACAATCCGGAAGCGACCAACGCCTTGATTGACAAGGATGGATGGCTACATTCTGGAGACATAGCTTACTGGGACGAAGACGAACACTTCTTCATCGTTGACCGCCTGAAGTCTCTGATTAAGTACAAAGGCTATCAGGTGGCTCCCGCTGAATTGGAATCCATCTTGCTCCAACACCCCAACATCTTCGACGCAGGTGTCGCAGGTCTTCCCGACGATGACGCCGGTGAACTTCCCGCCGCCGTTGTTGTTTTGGAGCACGGAAAGACGATGACGGAAAAAGAGATCGTGGATTACGTCGCCAGTCAAGTAACAACCGCGAAAAAGTTGCGCGGAGGAGTTGTGTTTGTGGACGAAGTACCGAAAGGTCTTACCGGAAAACTCGACGCAAGAAAAATCAGAGAGATCCTCATAAAGGCCAAGAAGGGCGGAAAGATCGCCGTGTAAttctagagtcgacctgcaggcatgcaagctgatccggctgctaacaaagcccgaaaggaagctgagttggctgctgccaccgctgagcaataactagcataaccccttggggcggccgcttcgagcagacatgataagatacattgatgagtttggacaaaccacaactagaatgcagtgaaaaaaatgctttatttgtgaaatttgtgatgctattgctttatttgtaaccattataagctgcaataaacaagttaacaacaacaattgcattcattttatgtttc

**The "Golden TAL Technology" toolbox**

TAL proteins with a designed repeat composition were constructed using a technology that is based on golden gate cloning [1,2]. Briefly, a library of single repeats was constructed with specific flanking *Bpi*I sites. Up to six repeats were assembled into a repeat module. One-to-four repeat modules are then assembled together with N- and C-terminus of Hax3 and an N-terminal GFP-tag into an expression vector.

**Part I - The repeat library**

Four key TAL repeat types were chosen to specifically recognize the four bases of the DNA. The specificities are, NI=A, HD=C, NN=G and A, NG=T. Single repeats were amplified by PCR from single-repeat templates [3] to add flanking *Bpi*I sites and cloned via cut-ligation into *Sma*I of pGGC, a pUC57-derivative with mutated *Bsa*I site. The specific *Bpi*I overhangs position each repeat of the library specifically within a six-repeat array. Therefore, the library consists of 24 individual repeat constructs.

Repeat library

*Bpi*I recognition sites (GAAGAC) are underlined and matching *Bpi*I-generated overhangs are shaded in similar colors (a: light grey, b: red, c: yellow, d: green, e: blue, f: purple, g: dark grey; see Fig. 3). Shaded in light grey and dark grey are the specific overhangs that match to the left and right side of the assembly vectors (see next chapter), respectively. Repeat sequences are only shown in part with translations in frame. The *Bpi*I overhangs were chosen to be highly different to avoid false ligation products. This was accomplished by using different codons for repeat amino acids and changing the position of the cut site. The sixth repeat is cloned as a half-repeat, because the last repeat in the repeat domain of a TAL is only a half-repeat.

*Typical repeat sequence*

LTPEQVVAIAS**xx**GGKQALETVQRLLPVLCQAHG

**xx** indicate the specificity-determining hypervariable residues (e.g. NI, HD, NN, NG).

*Repeat 1*

TTTGAAGACTTTTACCCCGGAGCAGGTGGTGGCCATCGCCAGCAATATTGGTGGCAAGCAGGCGCTGGAGACGGTGCAGCGGCTGTTGCCGGTGCTGTGCCAGGCCCATGGCCTGAAAGTCTTCAAA

*Repeat 2*

TTTGAAGACTTCTGACCCCGGAGCAGGTGGTGGCCATCGCCAGCAATATTGGTGGCAAGCAGGCGCTGGAGACGGTGCAGCGGCTGTTGCCGGTGCTGTGCCAGGCCCATGGCCTGACACCGAAGTCTTCAAA

*Repeat 3*

TTTGAAGACTTACCGGAGCAGGTGGTGGCCATCGCCAGCAATATTGGTGGCAAGCAGGCGCTGGAGACGGTGCAGCGGCTGTTGCCGGTGCTGTGCCAGGCCCATGGCCTCACCAAGTCTTCAAA

*Repeat 4*

TTTGAAGACTTCACCCCGGAGCAGGTGGTGGCCATCGCCAGCAATATTGGTGGCAAGCAGGCGCTGGAGACGGTGCAGCGGCTGTTGCCGGTGCTGTGCCAGGCCCATGGCCTGACTCAAGTCTTCAAA

*Repeat 5*

TTTGAAGACTTACTCCGGAGCAGGTGGTGGCCATCGCCAGCAATATTGGTGGCAAGCAGGCGCTGGAGACGGTGCAGCGGCTGTTGCCGGTGCTGTGCCAGGCCCATAAGTCTTCAAA

*Repeat 6*

TTTGAAGACTTCCATGGCCTGACCCCGGAGCAGGTGGTGGCCATCGCCAGCAATATTGGTGGCAAGCAGGCGCTGGAAAGTCTTCAAA

**Part II - Stop repeats to clone repeat modules with less than six repeats**

Stop repeats specifically align with the repeat array with their left *Bpi*I overhang, and their right *Bpi*I overhang matches to the assembly vector. Thereby, they terminate the repeat array and repeat modules with less than six repeats can be constructed. The stop repeats were cloned with NG (specifying for T) and NS (specifying for A, C, G, and T) to allow flexibility at the last TAL box position.

*Stop repeat 1*

TTTGAAGACTTTTACCCCGGAGCAGGTGGTGGCCATCGCCAGCAATATTGGTGGCAAGCAGGCGCTGGAAAGTCTTCAAA

*Stop repeat 2*

TTTGAAGACTTCTGACCCCGGAGCAGGTGGTGGCCATCGCCAGCAATATTGGTGGCAAGCAGGCGCTGGAAAGTCTTCAAA

*Stop repeat 3*

TTTGAAGACTTACCGGAGCAGGTGGTGGCCATCGCCAGCAATATTGGTGGCAAGCAGGCGCTGGAAAGTCTTCAAA

*Stop repeat 4*

TTTGAAGACTTCACCCCGGAGCAGGTGGTGGCCATCGCCAGCAATATTGGTGGCAAGCAGGCGCTGGAAAGTCTTCAAA

*Stop repeat 5*

TTTGAAGACTTACTCCGGAGCAGGTGGTGGCCATCGCCAGCAATATTGGTGGCAAGCAGGCGCTGGAAAGTCTTCAAA

**Part III - Assembly vectors containing repeat modules**

One to six repeats modules were assembled using golden gate cloning into matching *Bpi*I sites of an assembly vector to constitute a repeat assembly. The assembly vector contains a kanamycin resistance gene to distinguish ligation products from the ampicillin-resistant repeat library. The inserted repeats replace a *lacZ* gene which is used for blue-white selection. The repeats are flanked by specific *Bsa*I sites that position the repeat assembly within the final TAL protein. Repeat assemblies with matching *Bsa*I sites can be ligated. One to four repeat assemblies corresponding to 0.5 to 23.5 repeats are inserted into a TAL protein.

Part of the assembly vector sequences are shown. *Bsa*I recognition sites (GGTCTC) are in black bold face and nucleotides of matching *Bsa*I overhangs are bold face in similar colors (L: light grey, A: red, B: green, C: blue, R: dark grey; see Fig. 3). The *Bsa*I sites "L" (left, light grey) and "R" (right, dark grey) match to the N-terminus and C-terminus of the TAL protein, respectively. *Bpi*I recognition sites are underlined and *Bpi*I overhangs matching to the first and last repeat of the repeat assembly are shaded in light and dark grey, respectively. Relevant codons are translated. Repeat assemblies that are not the last one in the repeat array (i.e. *Bsa*I sites A, B, or C on the right side) are complemented with a half repeat from the assembly vector to generate a full repeat.

*Assembly vector L-A*

GTC**GGTCTC**A**GAAC**CTTACAAGTCTTCCTT•••*lacZ*•••

L T

•••GGCGAAGACTTTGGAGACGGTGCAGCGGCTGTTGCCGGTGCTGTGCCAGG**CGCA**T**GAGACC**CAC

L E T V Q R L L P V L C Q A H

*Assembly vector A-B*

GTC**GGTCTC**A**CGCA**TGGCCTTACAAGTCTTCCTT•••*lacZ*•••

H G L T

•••GGCGAAGACTTTGGAGACGGTGCAGCGGCTGTTGCCGGTGCTGTGC**CAGG**T**GAGACC**CAC

L E T V Q R L L P V L C Q

*Assembly vector B-C*

GTC**GGTCTC**A**CAGG**CGCATGGCCTTACAAGTCTTCCTT•••*lacZ*•••

Q A H G L T

•••GGCGAAGACTTTGGAGACGGTGCAGCGGCTGTTGCCGGTGCT**GTGC**T**GAGACC**CAC

L E T V Q R L L P V L C

*Assembly vector C-R*

GTC**GGTCTC**A**GTGC**CAGGCGCATGGCCTTACAAGTCTTCCTT•••*lacZ*•••

C Q A H G L T

•••GGCGAAGACTTTGGAG**AGCA**T**GAGACC**CAC

L E S

*Assembly vector A-R*

GTC**GGTCTC**A**CGCA**TGGCCTTACAAGTCTTCCTT•••*lacZ*•••

H G L T

•••GGCGAAGACTTTGGAG**AGCA**T**GAGACC**CAC

L E S

*Assembly vector B-R*

GTC**GGTCTC**A**CAGG**CGCATGGCCTTACAAGTCTTCCTT•••*lacZ*•••

Q A H G L T

•••GGCGAAGACTTTGGAG**AGCA**T**GAGACC**CAC

L E S

*Complete sequence of assembly vector L-A*

The position of the *BsaI* sites is indicated in bold face type. The other assembly vectors can be deduced from the L-A vector by substituting the region of the *BsaI* and *BpiI* sites as indicated above.

TGACTCGCTGCGCTCGGTCGTTCGGCTGCGGCGAGCGGTATCAGCTCACTCAAAGGCGGTAATACGGTTATCCACAGAATCAGGGGATAACGCAGGAAAGAACATGTGAGCAAAAGGCCAGCAAAAGGCCAGGAACCGTAAAAAGGCCGCGTTGCTGGCGTTTTTCCATAGGCTCCGCCCCCCTGACGAGCATCACAAAAATCGACGCTCAAGTCAGAGGTGGCGAAACCCGACAGGACTATAAAGATACCAGGCGTTTCCCCCTGGAAGCTCCCTCGTGCGCTCTCCTGTTCCGACCCTGCCGCTTACCGGATACCTGTCCGCCTTTCTCCCTTCGGGAAGCGTGGCGCTTTCTCAATGCTCACGCTGTAGGTATCTCAGTTCGGTGTAGGTCGTTCGCTCCAAGCTGGGCTGTGTGCACGAACCCCCCGTTCAGCCCGACCGCTGCGCCTTATCCGGTAACTATCGTCTTGAGTCCAACCCGGTAAGACACGACTTATCGCCACTGGCAGCAGCCACTGGTAACAGGATTAGCAGAGCGAGGTATGTAGGCGGTGCTACAGAGTTCTTGAAGTGGTGGCCTAACTACGGCTACACTAGAAGGACAGTATTTGGTATCTGCGCTCTGCTGAAGCCAGTTACCTTCGGAAAAAGAGTTGGTAGCTCTTGATCCGGCAAACAAACCACCGCTGGTAGCGGTGGTTTTTTTGTTTGCAAGCAGCAGATTACGCGCAGAAAAAAAGGATCTCAAGAAGATCCTTTGATCTTTTCTACGGGGTCTGACGCTCAGTGGAACGAAAACTCACGTTAAGGGATTTTGGTCATGAGATTATCAAAAAGGATCTTCACCTAGATCCTTTTAAATTAAAAATGAAGTTTTAAATCAATCTAAAGTATATATGAGTAAACTTGGTCTGACAGAGTGTTTGAAGCAAAAGTGTTAGAAAAACTCATCGAGCATCAAATGAAACTGCAATTTATTCATATCAGGATTATCAATACCATATTTTTGAAAAAGCCGTTTCTGTAATGAAGGAGAAAACTCACCGAGGCAGTTCCATAGGATGGCAAGATCCTGGTATCGGTCTGCGATTCCGACTCGTCCAACATCAATACAACCTATTAATTTCCCCTCGTCAAAAATAAGGTTATCAAGTGAGAAATCACCATGAGTGACGACTGAATCCGGTGAGAATGGCAAAAGTTTATGCATTTCTTTCCAGACTTGTTCAACAGGCCAGCCATTACGCTCGTCATCAAAATCACTCGCATCAACCAAACCGTTATTCATTCGTGATTGCGCCTGAGCGAGACGAAATACGCGATCGCTGTTAAAAGGACAATTACAAACAGGAATCGAATGCAACCGGCGCAGGAACACTGCCAGCGCATCAACAATATTTTCACCTGAATCAGGATATTCTTCTAATACCTGGAATGCTGTTTTTCCGGGGATCGCAGTGGTGAGTAACCATGCATCATCAGGAGTACGGATAAAATGCTTGATGGTCGGAAGAGGCATAAATTCCGTCAGCCAGTTTAGTCTGACCATCTCATCTGTAACATCATTGGCAACGCTACCTTTGCCATGTTTCAGAAACAACTCTGGCGCATCGGGCTTCCCATACAAGCGATAGATTGTCGCACCTGATTGCCCGACATTATCGCGAGCCCATTTATACCCATATAAATCAGCATCCATGTTGGAATTTAATCGCGGCCTCGACGTTTCCCGTTGAATATGGCTCATAACACCCCTTGTATTACTGTTTATGTAAGCAGACAGTTTTATTGTTCATGATGATATATTTTTATCTTGTGCAATGTAACATCAGAGATTTTGAGACACGGGCCAGAGCTGCCAGGAAACAGCTATGACCATGTAATACGACTCACTATAGGGGATATCAGCTGGGTC**GGTCTC**AGAACCTTACAAGTCTTCCTTGTCTGTAAGCGGATGCCGGGAGCAGACAAGCCCGTCAGGGCGCGTCAGCGGGTGTTGGCGGGTGTCGGGGCTGGCTTAACTATGCGGCATCAGAGCAGATTGTACTGAGAGTGCACCATATGCGGTGTGAAATACCGCACAGATGCGTAAGGAGAAAATACCGCATCAGGCGCCATTCGCCATTCAGGCTGCGCAACTGTTGGGAAGGGCGATCGGTGCGGGCCTCTTCGCTATTACGCCAGCTGGCGAAAGGGGGATGTGCTGCAAGGCGATTAAGTTGGGTAACGCCAGGGTTTTCCCAGTCACGACGTTGTAAAACGACGGCCAGTGAATTCGAGCTCGGTACCTCGCGAATGCATCTAGATATCGGATCCCGGGCCCGTCGACTGCAGAGGCCTGCATGCAAGCTTGGCGTAATCATGGTCATAGCTGTTTCCTGTGTGAAATTGTTATCCGCTCACAATTCCACACAACATACGAGCCGGAAGCATAAAGTGTAAAGCCTGGGGTGCCTAATGAGTGAGCTAACTCACATTAATTGCGTTGCGCTCACTGCCCGCTTTCCAGTCGGGAAACCTGTCGTGCCAGCTGCATTAATGAATCGGCCAACGCGCGGGGAGAGGCGGTTTGCGTATTGGGCGAAGACTTTGGAGACGGTGCAGCGGCTGTTGCCGGTGCTGTGCCAGGCGCAT**GAGACC**CAC

**Part IV - Other golden gate modules**

The *Bsa*I recognition sites are in bold face, and the overhangs are shaded in grey. Relevant codons are translated.

*GFP-tag*

*gfp* was subcloned into pGGC with flanking *Bsa*I sites to generate the following overhangs.

TTT**GGTCTC**GATCCATG•••*gfp*•••CATATGA**GAGACC**AAA

M H M

*N-terminus Hax3*

The first 864 nucleotides of *hax3* were subcloned into pGGC with flanking *Bsa*I sites.

TTT**GGTCTC**ATATGGATCCC•••N-terminus Hax3•••CCCCTGAACT**GAGACC**AAA

M D P P L N

*C-terminus Hax3*

The last 837 nucleotides of *hax3* were subcloned into pGGC with flanking *Bsa*I sites.

TTT**GGTCTC**AAGCATTGTT•••C-terminus Hax3•••CCTCAGTGAAGCTTT**GAGACC**AAA

S I V P Q *

**Part V - Expression vectors**

Expression vectors are designed to accomodate all other modules and place them under control of a strong promoter.

*Expression in planta*

The cauliflower mosaic virus *35S* promoter drives expression of the *TAL*-derivative gene *in planta*. The backbone of the vector was derived from pBGWFS7 (4). Insertion of the *Bsa*I fragments of the *TAL* gene replaced a *ccdB* cassette used for negative selection of false ligation products. The *Bsa*I recognition sites are in bold face, and the overhangs are shaded in grey.

GGATCCA**GAGACC**•••*ccdB*•••**GGTCTC**AGCTT

*Expression in human cells*

The cytomegalovirus *CMV* promoter drives expression of the *TAL*-derivative gene in human cells. A golden gate cassette containing a *lacZ* with flanking *Bsa*I sites was inserted into a pcDNA5 derivative with mutated *Bsa*I sites. The following *Bsa*I overhangs were used:

GGATCCT**GAGACC**•••*lacZ*•••**GGTCTC**AGCTT

**Promoter regions of human genes chosen as targets for TAL protein-directed expression.**

A partial region of the promoters is shown. Uppercase letters: coding region; lowercase letters: untranslated regions; red font: 5'UTR regions; underlined font: ATG start codon.

*PUMA* beta (BBC3; GenBank: AF354655.1)

ggagatttcacgtgagatatagattacctgcatctcttgggggagctaagagtgtg**tacttggaggcagtcaagt**ttgagaagtctgacatccttactcagccagccccacactaggcactggaaggtgagtcactctggtgaggcgattgcgattgggtgagacccagtaaggatggaaagtgtagaggagacaggaatccacggctttggaaaaaggaaggacaaaactcaccaaaccagagcagggcaggaagtaacaatgagaaactgaaaaagaaacggaatggaaagctatgagacaggATGAAATTTGGCATGGGGTCTGCCCAGGCATGTCCATGCCAGGTG

*INFα1* (GenBank: AF354655.1)

cggctctaaactcatgtaaagagtgcatgaaggaaagcaaaaacagaaa**tggaaagtggcccagaagcat**taagaaagtggaaatcagtatgttccctatttaaggcatttgcaggaagcaaggccttcagagaacctagagcccaaggttcagagtcacccatctcagcaagcccagaagtatctgcaatatctacgATGGCCTCGCCCTTTGCTTTACTGATGGTCCTGGTGGTGCTCAGCTGCAAGT

*INFβ* (GenBank: NM_002176.2)

acataggaaaactgaaagggagaagtgaaagtgggaaattcctctgaatagagagaggacca**tctcatataaataggccat**acccatggagaaaggacattctaactgcaacctttcgaagcctttgctctggcacaacaggtagtaggcgacactgttcgtgttgtcaacATGACCAACAAGTGTCTCCTCCAA

**Supplemental References**

1. Engler C, Gruetzner R, Kandzia R, & Marillonnet S (2009) Golden gate shuffling: a one-pot DNA shuffling method based on type IIs restriction enzymes. *PLoS ONE* 4(5):e5553.

2. Engler C, Kandzia R, & Marillonnet S (2008) A one pot, one step, precision cloning method with high throughput capability. *PLoS ONE* 3(11):e3647.

3. Boch J*, et al.* (2009) Breaking the code of DNA binding specificity of TAL-type III effectors. *Science* 326(5959):1509-1512.

4. Karimi M, De Meyer B, & Hilson P (2005) Modular cloning and expression of tagged fluorescent protein in plant cells. *Trends Plant Sci.* 10(3):103-105.
