# Supplementary figures and images for: Transcriptional Activators of Human Genes with Programmable DNA-Specificity
Source: PLoS One. 2011 May 19;6(5):e19509. doi: 10.1371/journal.pone.0019509 (PMC3098229; doi:10.1371/journal.pone.0019509)

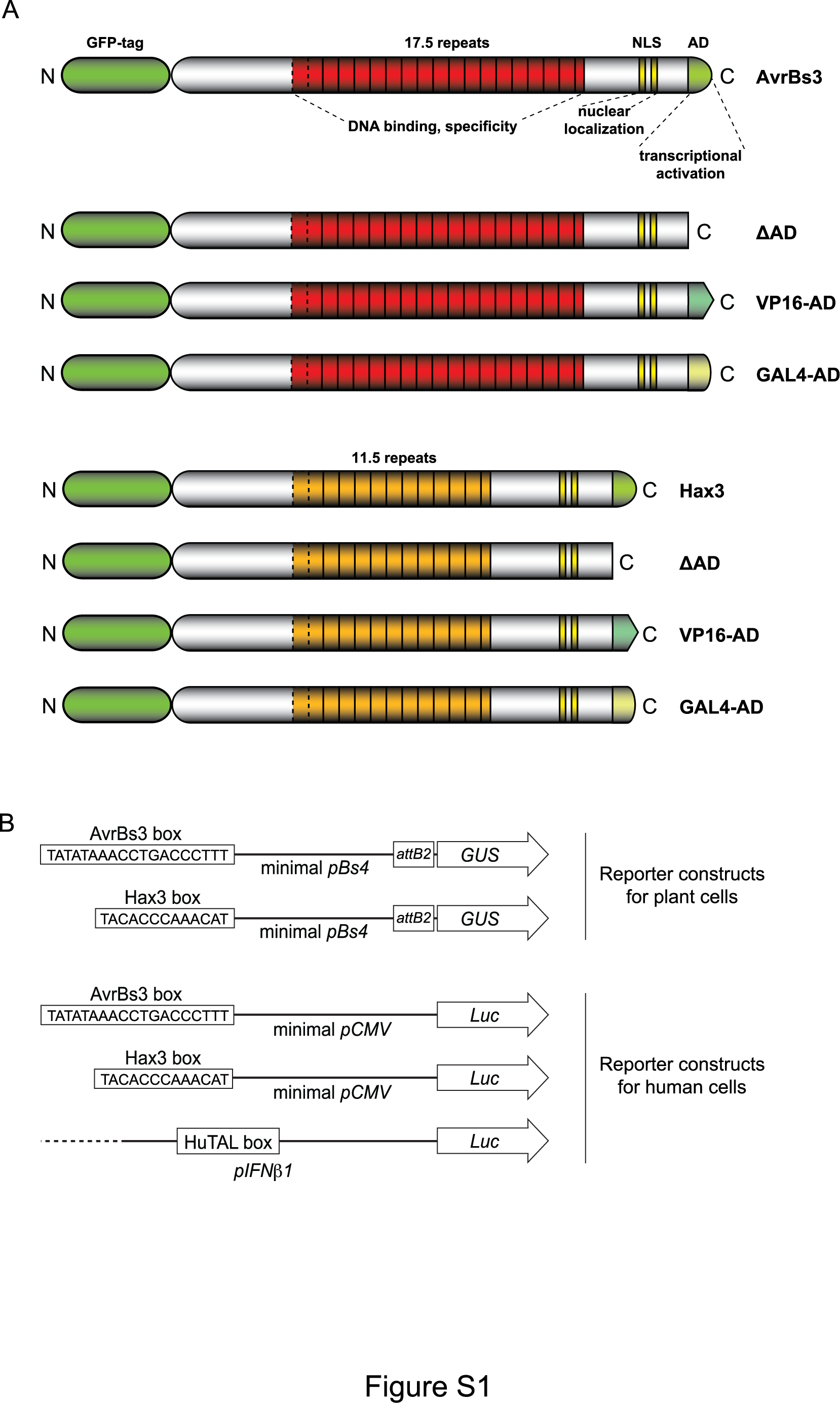

Supplement: Figure S1 — TAL effector-derivatives and reporter constructs. (A) Functional domains of TAL effector-derivatives used. The endogenous C-terminal transcriptional activation domain (AD; C-terminal 31 aa) was deleted or replaced by the ADs from herpes simplex virus VP16 (C-terminal 68 aa) or yeast GAL4 (C-terminal 113 aa). NLS: nuclear localization signals; red: repeat domain of AvrBs3 and derivatives; yellow: repeat domain of Hax3 and derivatives. All constructs carried an N-terminal green fluorescent protein (GFP) tag. (B) Reporter constructs used for transient expression in plant and human cells, respectively. For experiments in plants, TAL protein target DNA boxes were inserted in front of a minimal Bs4 promoter which has low basal activity upstream of a promoterless uidA (GUS) reporter gene as described [4]. For studies with human cells, TAL protein target DNA boxes were placed upstream of the minimal CMV promoter and a promoterless luciferase (luc) gene into a pF12A RM Flexi (Promega) reporter vector. To study activity of the endogenous interferon β promoter, the minimal CMV promoter was replaced by part of the INFβ promoter. (TIF) [file pone.0019509.s002.tif]

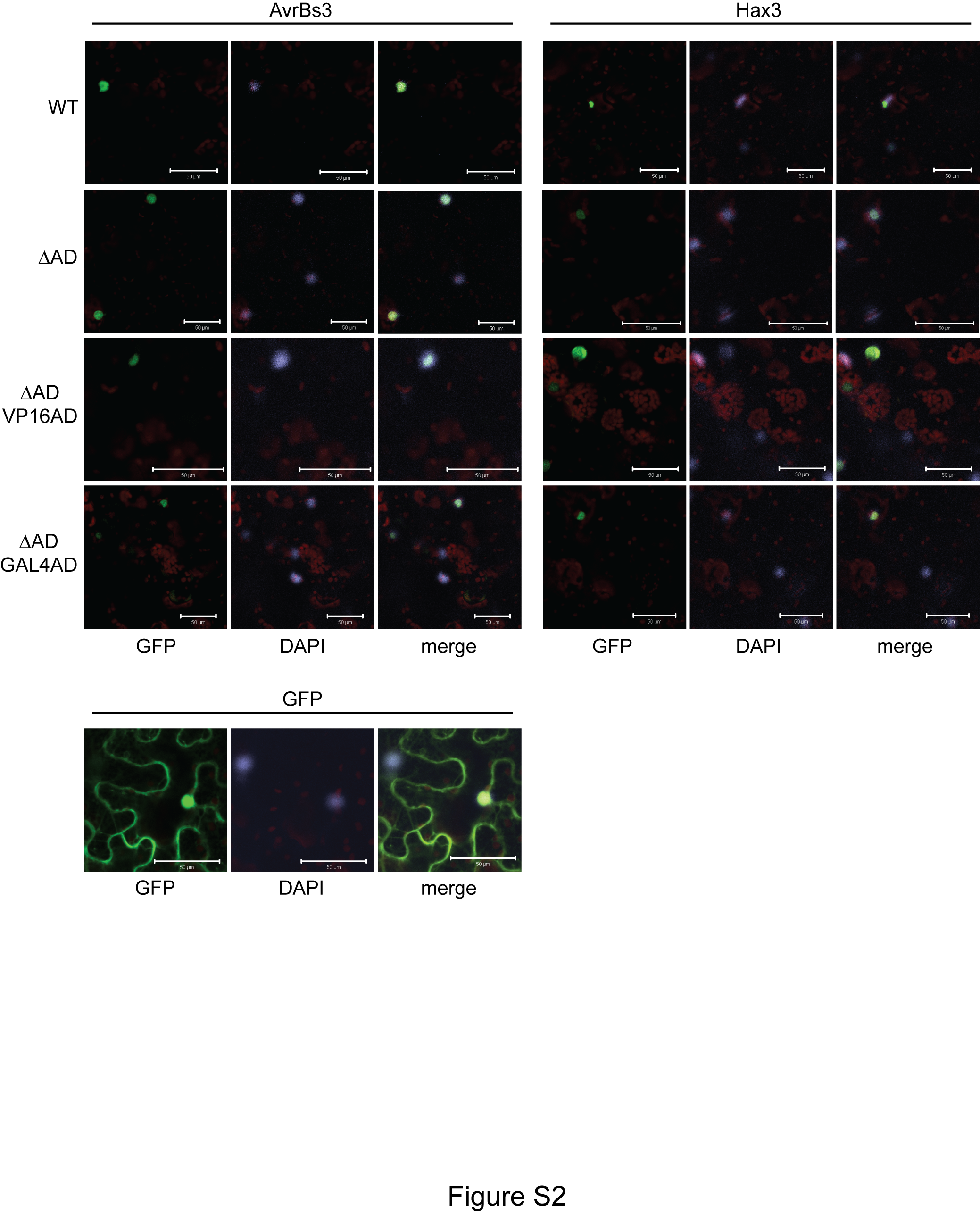

Supplement: Figure S2 — GFP-TAL protein fusions localized to the plant nucleus. GFP-TAL protein fusions or GFP alone were expressed transiently in leaf cells of Nicotiana benthamiana via Agrobacterium-mediated delivery. Two days post infiltration, N. benthamiana leaf epidermis cells were stained with DAPI (4′,6-diamidino-2-phenylindole) and analyzed by confocal laser scanning microscopy. Green: GFP fluorescence; blue: DAPI fluorescence; red: chlorophyll autofluorescence. Scale bars indicate 50 µm. (TIF) [file pone.0019509.s003.tif]

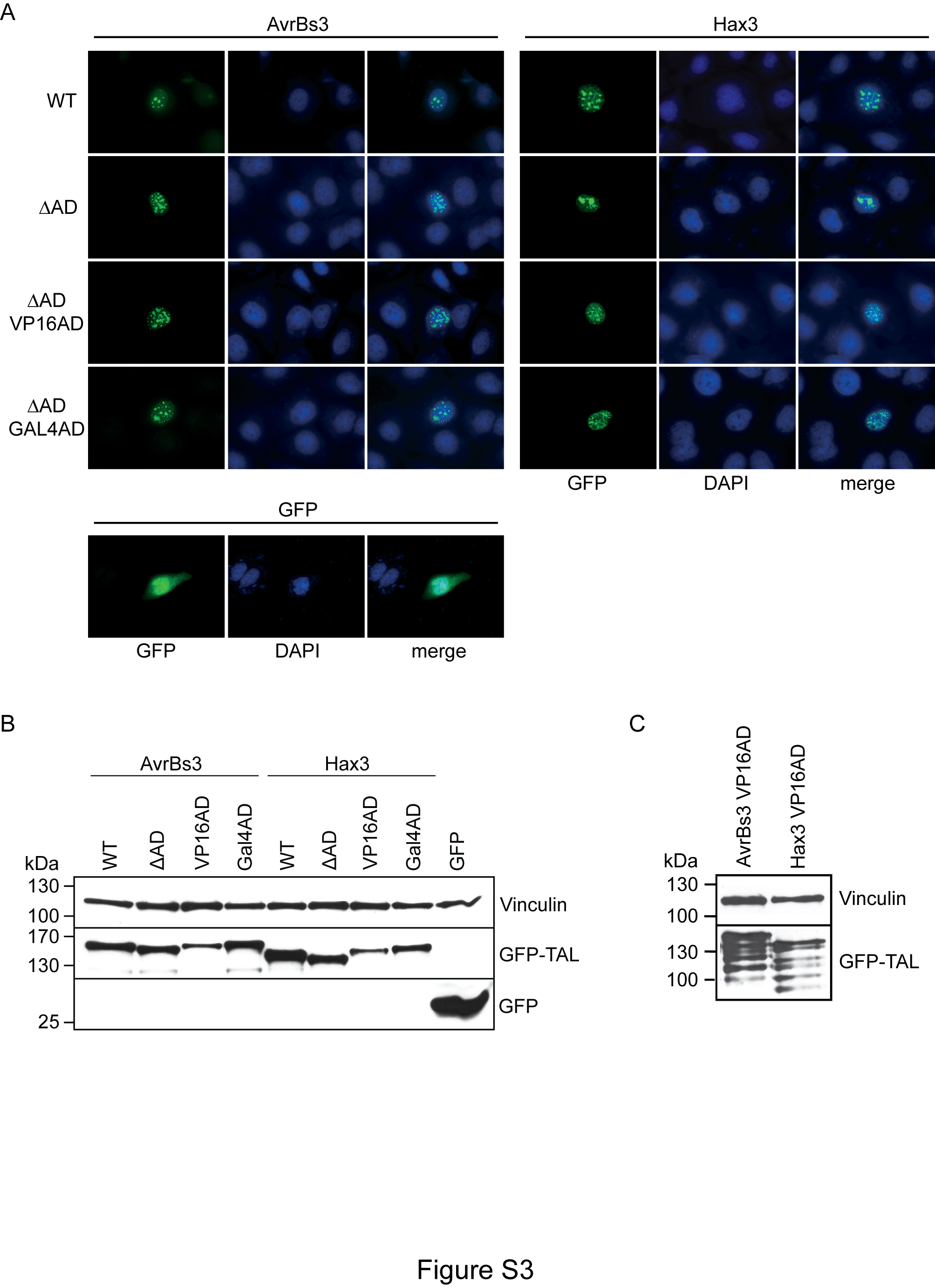

Supplement: Figure S3 — GFP-TAL protein fusions are expressed and localize to the nucleus in human cells. (A) GFP-TAL protein fusions or GFP alone were expressed after transfection of human Hek293T-Rex cells. One day post transfection, cells were stained with DAPI (4′,6-diamidino-2-phenylindole) and analyzed by fluorescence microscopy. Green: GFP fluorescence; blue: DAPI fluorescence. (B) Western-blot analysis of whole cell extracts of transfected Hek293T-Rex cells. (C) TAL protein-derivatives expressed in HeLa cells show degradation patterns. (B+C) GFP and GFP-TAL protein fusions were detected using α-GFP antibody. An α-Vinculin antibody was used as constitutive loading control. (TIF) [file pone.0019509.s004.tif]

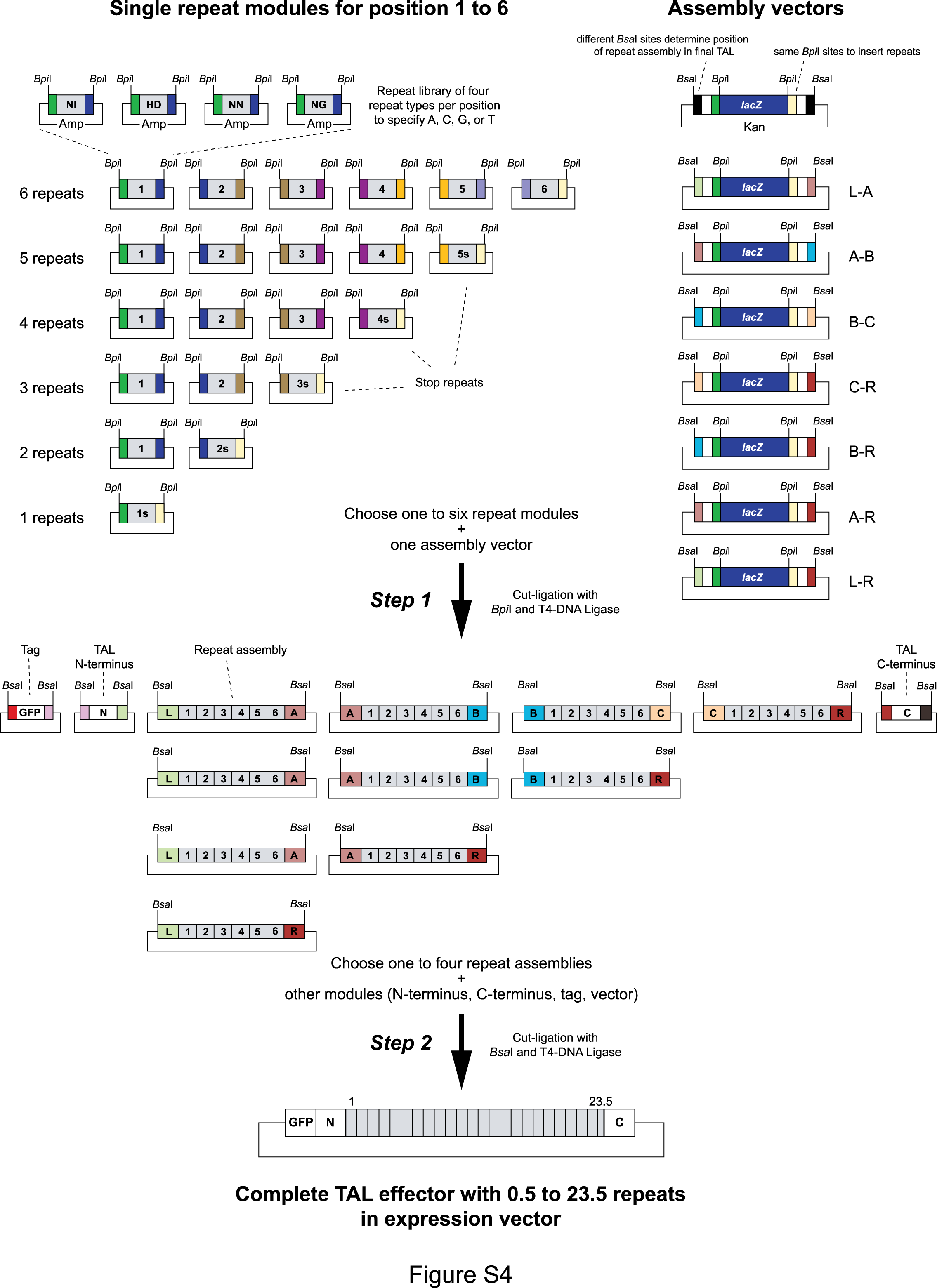

Supplement: Figure S4 — Golden TAL Technology toolbox. Single repeat modules were subcloned with flanking BpiI sites that specify their position in the repeat array. A library of four repeat types (NI = A, HD = C, NN = G/A, NG = T) was constructed for each of the six repeat positions. Stop repeats (1s to 5s) can be used to terminate the repeat assembly. In cloning step 1, up to six repeats are inserted into an assembly vector to generate a repeat assembly. Different assembly vectors are used to position the repeat assembly within the final TAL protein repeat domain. In cloning step 2, individual repeat assemblies, Hax3 N- and C-termini, and an N-terminal GFP-tag are ligated into an expression vector to generate the complete TAL gene. (TIF) [file pone.0019509.s005.tif]

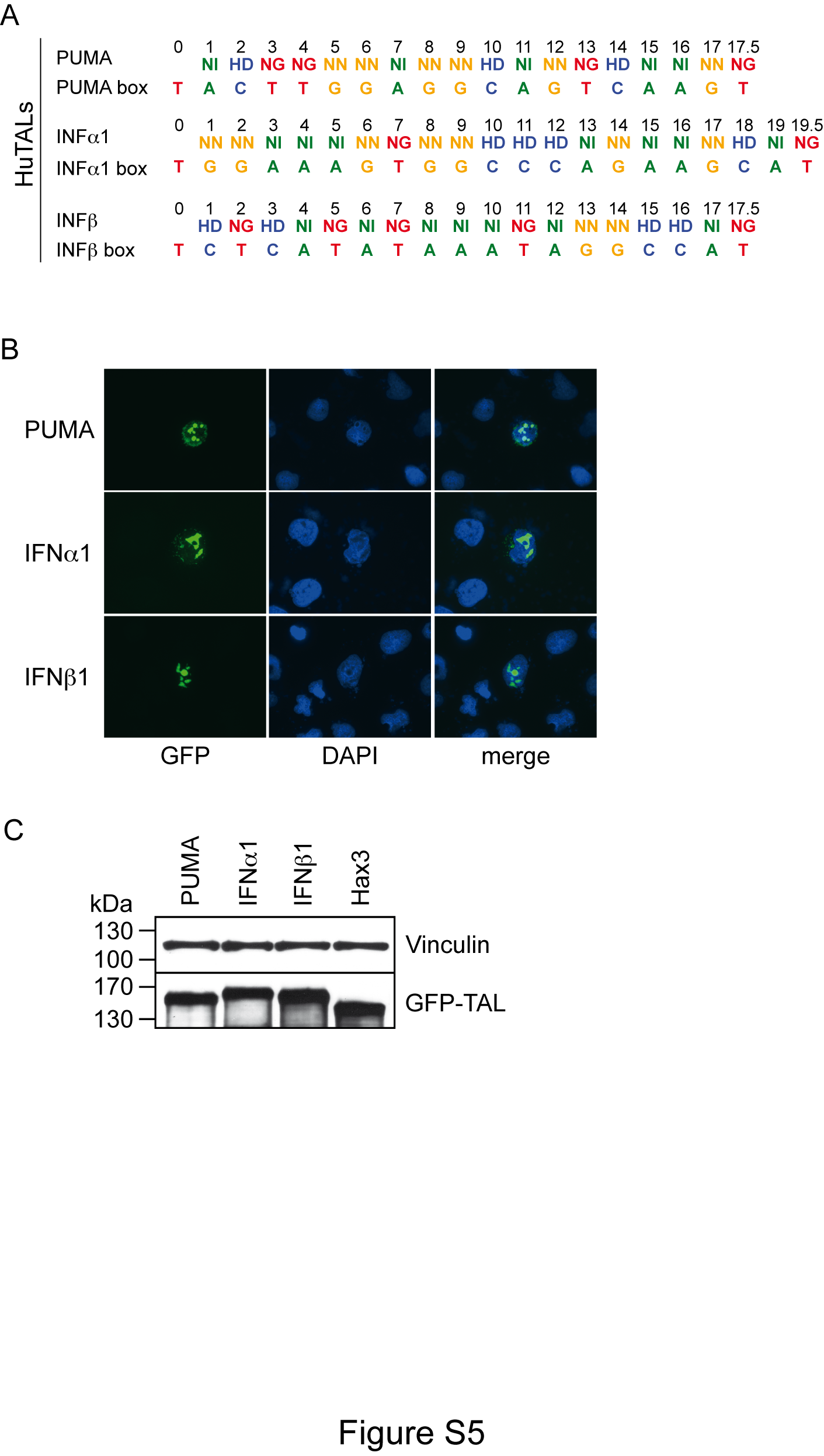

Supplement: Figure S5 — HuTALs designed to target expression of human genes. (A) HuTALs with 17.5 to 19.5 repeats matching a DNA sequence (box) in the promoter region of target genes are assembled as N-terminal GFP fusions using the “Golden TAL technology”. (B) GFP-TAL protein fusions were expressed after transfection of human Hek293T-Rex cells. One day post transfection, cells were stained with DAPI (4′,6-diamidino-2-phenylindole) and analyzed by fluorescence microscopy. Green: GFP fluorescence; blue: DAPI fluorescence. (C) Western-blot analysis of whole cell extracts of Hek293T-Rex cells transfected with HuTAL expression constructs. (TIF) [file pone.0019509.s006.tif]
