# Supplementary material for: Transcriptional Activators of Human Genes with Programmable DNA-Specificity
Source: PLoS One. 2011 May 19;6(5):e19509. doi: 10.1371/journal.pone.0019509 (PMC3098229; doi:10.1371/journal.pone.0019509)
Supplement: Table S1 — Selected primers used in this study. (DOC) [file pone.0019509.s007.doc]

**Table S1.** Selected primers used in this study.

| **Name, Purpose** | **Nucleotide sequence** |
| --- | --- |
| **Cloning of *INFβ1* promoter** | |
| IFNbProfw | TGCTCTAGATCCCGCTGCCTCCACAGATACC |
| IFNbProrv | CCCAAGCTTGTTGACAACACGAACAGTGTCGCC |
|  |  |
| **RT-PCR** | |
| IFNalpha1afw | CTTATTGACTCATACACCAGGTCACGC |
| IFNalpha1arv | GCATGGTCATAGTTATAGCAGGGGTG |
| IFNbetafw | GACTTACAGGTTACCTCCGAAACTG |
| IFNbetarv | TGAAGCAATTGTCCAGTCCCAG |
| Pumafw | CCCCATCAATCCCATTGCATAGG |
| Pumarv | GGCTTCAGCCAAAATCTCCCAC |
|  |  |
| **Cloning of TAL repeats** | |
| 1-F | TTTGAAGACTTTTACCCCGGAGCAGGTGGTGGCC |
| 1-R | TTTGAAGACTTTCAGGCCATGGGCCTGGCACAGCAC |
| 2-F | TTTGAAGACTTCTGACCCCGGAGCAGGTGGTG |
| 2-R | TTTGAAGACTTCGGTGTCAGGCCATGGGCCTGGCACAGCAC |
| 3-F | TTTGAAGACTTACCGGAGCAGGTGGTGGCCATCGCC |
| 3-R | TTTGAAGACTTGGTGAGGCCATGGGCCTGGCACAGCAC |
| 4-F | TTTGAAGACTTCACCCCGGAGCAGGTGGTGGCC |
| 4-R | TTTGAAGACTTGAGTCAGGCCATGGGCCTGGCACAGCAC |
| 5-F | TTTGAAGACTTACTCCGGAGCAGGTGGTGGCCATC |
| 5-R | TTTGAAGACTTATGGGCCTGGCACAGCACCGG |
| 6-F | TTTGAAGACTTCCATGGCCTGACCCCGGAGCAGGTGG |
| 6III-R | TTTGAAGACTTTCCAGCGCCTGCTTGCC |
|  |  |
